# Poll Everywhere to Encourage Learner Satisfaction and Participation in Internal Medicine Fellowship Didactics

**DOI:** 10.7759/cureus.7078

**Published:** 2020-02-22

**Authors:** Sonia Castillo, Laura Thomas, Sri Yarlagadda, Yousuf Ahmed, Jessica R Newman

**Affiliations:** 1 Pulmonary/Critical Care Medicine, Kansas City Veteran Affairs (VA) Medical Center, Kansas City, USA; 2 Internal Medicine, University of Kansas Medical Center, Kansas City, USA; 3 Pulmonology/Critical Care, University of Kansas Medical Center, Kansas City, USA; 4 Nephrology, University of Kansas Medical Center, Kansas City, USA; 5 Infectious Diseases, University of Kansas Medical Center, Kansas City, USA

**Keywords:** medical education, didactics, audience response, interactive learning

## Abstract

Active learning improves self-reported engagement and satisfaction in medical education. Audience response systems are one mechanism of encouraging participation, especially in a setting in which learners in varying educational levels are present. Three fellowships participated in this educational quality improvement project where Poll Everywhere® was incorporated into didactics. Attendees were invited to complete a 4-question retrospective pre-post satisfaction survey. Incorporation of the Poll Everywhere® audience response system resulted in a shift in more favorable satisfaction scores and self-perceived attentiveness compared to the pre-intervention responses.

## Introduction

Adopting active learning techniques in higher education venues can result in significant improvement in self-reported student engagement and satisfaction scores as well as enhanced academic performance [1-5]. As more graduate medical education programs adopt active learning pedagogies into their didactics, engaging learners and maintaining participation is vital for helping learners assimilate information [6]. Utilizing small group sessions and establishing collegial methods of questioning may encourage participation. Audience response systems (ARS) are popular with learners, associated with retention, and are advantageous for boosting contribution in learning sessions, as responses can remain anonymous [7]. First-generation systems, such as “iClicker,” had the benefit of being provided by the course or instructor, alleviating planning on the part of the learner, and could eliminate concern for distraction with web-based programs. They also required more effort on the part of the instructor with pitfalls, including battery life, limited capabilities, and cost. Newer technologies, such as Poll Everywhere®, allow the audience to respond on the web, via an application on a smartphone or via SMS texting on their phone. While these systems may permit distractions, as the user may be on the web during the activity, they have multiple new features including multiple-choice questions, open response, live word clouds, clickable images, up and down-voting, and rank order items. Cell phones are readily available and considered beneficial for educational purposes by students, and phone/tablet applications for ARS are often free and can be downloaded quickly, permitting users a quick and straightforward platform for joining the educational opportunity [8-9]. The Poll Everywhere® ARS software functions in PowerPoint, Keynote, and Google Slides, and once the add-in software is installed on the presenter’s computer, a user sign-on at the start of the presentation opens the questions within the slide set for the response. The small size of each individual didactic session in our project permitted ease of helping new users to navigate downloading the application, which took seconds. While there are data supporting the use of ARS in larger and undergraduate educational venues, there is limited data on its effectiveness in smaller groups and graduate medical education settings. The objective was to improve self-reported learner engagement and satisfaction with Internal Medicine (IM) fellowship didactic sessions with the use of the Poll Everywhere® ARS.

## Materials and methods

This educational quality improvement project was performed at the University of Kansas Health System in Kansas City, KS, an academic hospital with over 750 staffed beds. Each IM subspecialty fellowship has an annual didactic schedule with a combination of lecture, case-based learning and journal club sessions. Three of the fellowships within the department of IM at the University of Kansas Health System participated in this project: Pulmonary and Critical Care Medicine (PCCM), Nephrology, and Infectious Diseases (ID), for a total of 21 fellows, with 18 fellows expected to attend weekly conferences at a given time (one PCCM fellow works night float and two ID fellows are off-site). The primary analysis of this project included the survey responses of attendees of the fellowship didactic sessions. Faculty, resident, and student learners present at the didactic sessions during the survey period were invited to participate. Learners were excluded from the analysis if they did not participate in the didactic sessions in which the anonymous paper survey was distributed. The sample size was determined based upon the number of attendees during the intervention didactic sessions. For the intervention, each divisional fellowship didactic schedule remained unchanged during the intervention period with exception of the addition of (a minimum of two) audience response prompts using the Poll Everywhere® software (San Francisco, California) during a two-week block from January 29 - February 9 of the 2017-2018 academic year. As the Poll Everywhere® software could be utilized by text messaging, smartphone, a tablet application, or SMS texting, this was introduced to learners before the intervention period via email, then again at the start of the session with information on logging on to respond. A four-question retrospective pre-post satisfaction survey was produced utilizing standardized five-item Likert scale responses and was disseminated to each attendee during each session of the second week of the intervention period. Each learner was surveyed only once. Questions addressed general satisfaction with the response system as well as the perception of attentiveness and self-assessed learning. A retrospective pre-post design allowed for respondents to have a better understanding of the intervention by experiencing it before evaluating the process. This permitted bias toward knowledge of the intervention. This project was reviewed and determined to be exempt by the University of Kansas Medical Center Human Subjects Committee (institutional review board).

## Results

**Figure 1:**
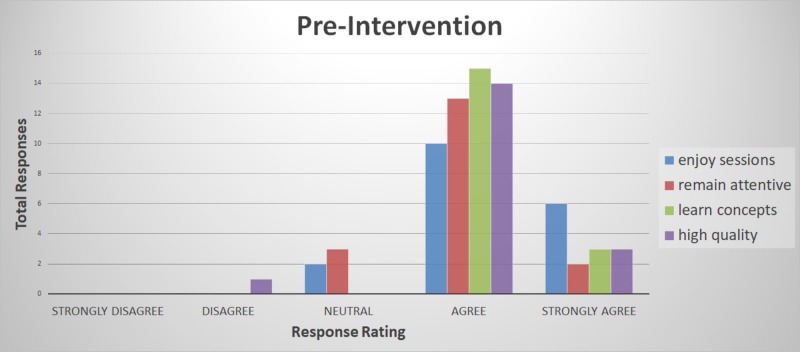
Pre-intervention survey responses

**Figure 2:**
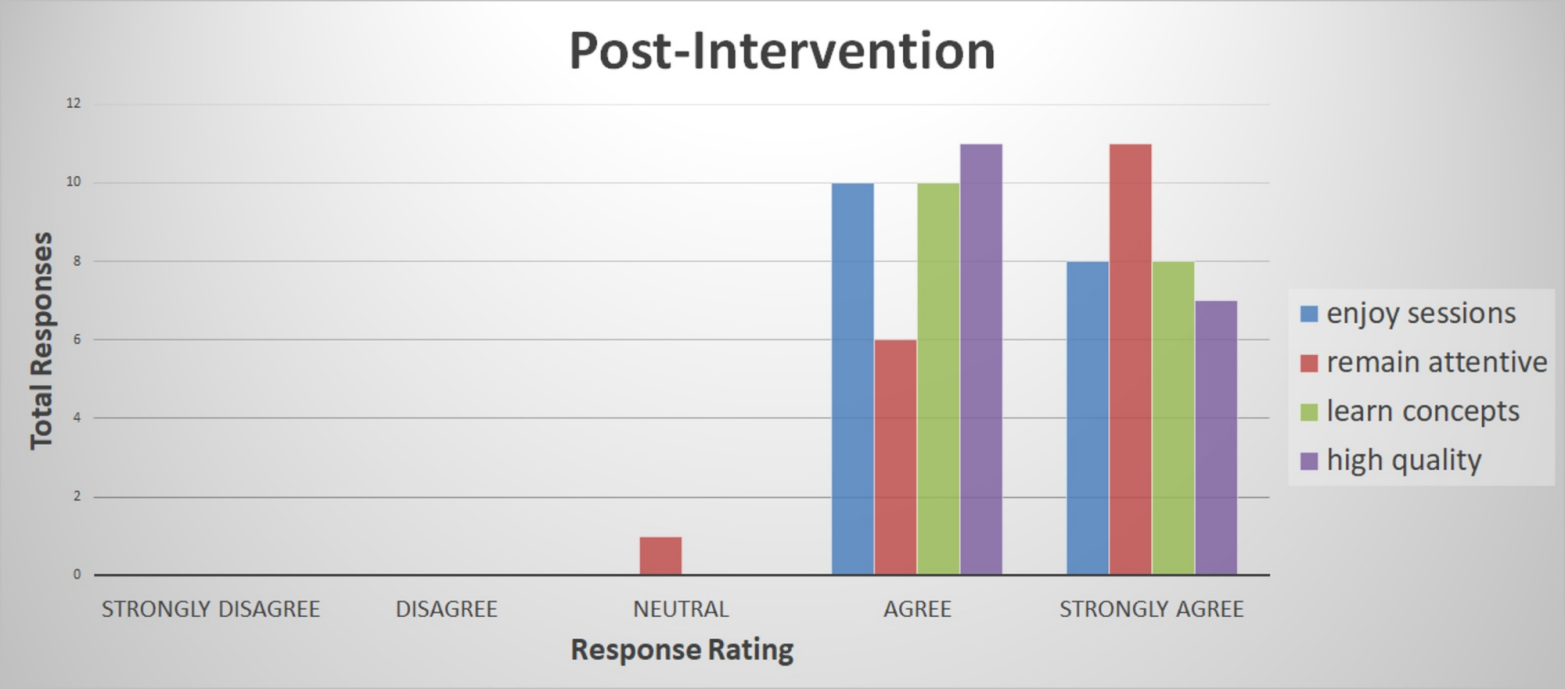
Post-intervention survey responses

The primary analysis of this project included attendees of the Internal Medicine Subspecialty Didactic sessions during the two-week intervention period. Responses were tabulated by total responses for each category of Likert item response. Fisher’s exact test was used to compare the difference between the favorable survey responses (agree and strongly agree in pre- and post-intervention groupings). Total responses in each category of the Likert scale response were tabulated in a Microsoft Excel spreadsheet (Microsoft Corporation, Redmond, Washington). The response rate among the fellows was 78% (14 out of 18 fellows). There were no “strongly disagree” responses for any survey question (Table 1, Figures 1-2). The Fisher exact test p-value was 0.0013.

**Table 1:** Pre-post satisfaction survey

Pre-Post Satisfaction Survey	
Q1	I enjoy attending fellowship didactic sessions
Q2	I remain attentive during fellowship didactic sessions
Q3	I learn new concepts and/or reinforce concepts I am familiar with during fellowship didactic sessions
Q4	Fellowship didactic sessions are of high quality

## Discussion

The benefits of incorporating active learning pedagogies into upper-level educational venues are well-known. Maintaining attentiveness and participation in these sessions is vital to promoting understanding and retention of material. Audience response systems allow for said participation and are favorably received in large, group microbiology lectures [10]. They have been shown to improve resident and attending satisfaction with smaller group didactics in radiology and emergency medicine as well as correlating to learning and retention in radiology, obstetrics, and family medicine; however, their utility in improving satisfaction with or self-perceived learning in IM or its subspecialties remains unclear [11-15]. Furthermore, the use of ARS for review quizzes has been shown to correlate to some residency specialty in-training exam scores, however, it remains unknown if the use of ARS would translate to improved IM board-subspecialty pass rates [16]. The Poll Everywhere® software specifically has not been evaluated in use in undergraduate or graduate medical education.

This study has several limitations. First, we surveyed learners at a single health system, which limits the generalizability of the findings, especially given the small sample size. Most of the survey responders were fellows, and thus the perceived benefit of this intervention for medical students or residents who attended the sessions could not be assessed uniquely. Additionally, in order to capture more learners (some of whom may have missed a didactic session during the survey week), we did not have an accurate count of all attendees at each individual didactic session and thus were not able to calculate an exact response rate for each didactic session in which the survey was administered. Additionally, there are limitations in linking the improved satisfaction and self-perceived learning scores with the intervention itself, given different educators were responsible for each didactic session, and changes in their presentation style may have contributed to score variation. As we asked that during each session, no changes were made in presentation style aside from the addition of the audience response questions, the improved scores suggest the intervention did relate to the improvement. Finally, satisfaction with and self-perceived learning is not necessarily correlated to measurable learning, though the utilization of Likert scales is preferred to post-decision wagering [17]. We did not repeat the survey at a later date to evaluate whether perceived satisfaction and learning were maintained.

## Conclusions

Our project demonstrates that graduate medical educational learners in three IM subspecialties had increased satisfaction with didactics after the incorporation of the Poll Everywhere system. Based on our retrospective pre-post survey results, we found a statistically significant increase in the number of strongly agree responses favoring the intervention. After the introduction of the Poll Everywhere ARS, there was a shift in favorable satisfaction scores, self-perceived learner attentiveness, and perceived learning benefit in fellowship didactics. Given this shift, audience response use will be continued within the intervention group IM subspecialty fellowship programs and encouraged in others. It is not clear if the increase in the utilization of active learning supporting technologies, such as ARS, would be able to generate objective measurable knowledge gains and retention in this graduate medical education group or whether such gains would, in turn, improve IM or IM subspecialty certification examination scores, and these are opportunities for further investigation.
